# From Data to Diagnosis: Building OSA Cohorts in Claims Data Linked to the National Health and Aging Trends Study

**DOI:** 10.1093/geroni/igaf122.576

**Published:** 2025-12-31

**Authors:** Jennifer Albrecht, Adam Spira, Alden Gross, Emerson M Wickwire, Halima Amjad, Atul Malhotra, Marcela Blinka, Christopher Kaufmann

**Affiliations:** University of Maryland, Baltimore, Maryland, United States; Johns Hopkins Bloomberg School of Public Health, Baltimore, Maryland, United States; Johns Hopkins Bloomberg School of Public Health, Baltimore, Maryland, United States; University of Maryland, Baltimore, Maryland, United States; Johns Hopkins Bloomberg School of Public Health, Baltimore, Maryland, United States; University of California, San Diego, San Diego, California, United States; Johns Hopkins University, Center on Aging and Health, Baltimore, Maryland, United States; University of Florida, Gainesville, Florida, United States

## Abstract

Evidence suggests obstructive sleep apnea (OSA) accelerates cognitive decline, yet data containing physician-assigned OSA diagnoses often lack granular measures of cognition and vice versa. Linking administrative claims to a longitudinal study with annual cognition measurement, such as the National Health and Aging Trends Study (NHATS), can strengthen these analyses. To determine the association between OSA and cognitive decline, we linked NHATS participants’ OSA diagnostic claims data to cognitive measures from NHATS study visits. We accessed NHATS data (2011-2021) linked to Medicare administrative claims (2008-2021) through NIA LINKAGE Enclave. Because patient-reported OSA diagnosis is not available in NHATS, we identified OSA by searching for ≥2 corresponding International Classification of Disease (ICD) codes from inpatient or outpatient Medicare claims. Main outcome of our analysis was cognitive performance; thus, we excluded those with pre-existing dementia (from NHATS algorithm or claims) at baseline. Finally, we matched people with OSA to those without. We identified 977 NHATS participants with OSA based on claims but without dementia. Half received the diagnosis before entering NHATS, making assignment of index date challenging. Further, participants with OSA were younger than those without, leading to difficulty matching. We assigned date of entry into NHATS as index date, assuming OSA was present the entire time, and matched participants without OSA to those with OSA based on age at NHATS entry and sex. In summary, we successfully identified NHATS participants with OSA and linked them to their cognitive data. Methodological challenges encountered during this process will be discussed.

